# Effects of ginsenoside Rb1 on second-degree burn wound healing and FGF-2/PDGF-BB/PDGFR-β pathway modulation

**DOI:** 10.1186/s13020-021-00455-w

**Published:** 2021-06-19

**Authors:** Li Zhang, Qin Hu, Haonan Jin, Yongzhao Yang, Yan Yang, Renhua Yang, Zhiqiang Shen, Peng Chen

**Affiliations:** School of Pharmaceutical Science & Yunnan Key Laboratory of Pharmacology for Natural Products, Kunming Medical University, 1168 West Chunrong Road, Chenggong, Kunming, Yunnan 650500 PR China

**Keywords:** Ginsenoside Rb1, Rats, Second-degree burn model, Burn wound healing, FGF-2, PDGF-BB/PDGFR-β

## Abstract

**Background:**

*Panax notoginseng* (Burk.) F. H. Chen (*P. notoginseng*) is a traditional Chinese medicine that has been used therapeutically for cardiovascular diseases, inflammatory diseases and traumatic injuries as well as for external and internal bleeding due to injury. Ginsenoside Rb1, a crucial monomeric active constituent extracted from *P. notoginseng*, has attracted widespread attention because of its potential anti-inflammatory, bacteriostatic, and cell growth-promoting effects. In this study, the therapeutic effects of ginsenoside Rb1 on second-degree burn in rats and the potential underlying mechanisms were explored.

**Methods:**

A rat model of second-degree burn injury was established, and skin wound healing was monitored at different time points after ginsenoside Rb1 treatment. HE staining was performed to identify burn severity, and biological tissues were biopsied on days 0, 7, 14, and 24 after treatment. Skin wound healing at different time points was monitored by macroscopic observation. Furthermore, IHC, WB, and RT-PCR were utilized to determine the protein and mRNA expression levels of PDGF-BB, PDGFR-β, and FGF-2 in wound tissues after treatment.

**Results:**

HE staining showed that after 24 days of ginsenoside Rb1 treatment, skin tissue morphology was significant improved. Macroscopic observation demonstrated that in ginsenoside Rb1-treated rats, the scab removal time and fur growth time were decreased, and the wound healing rate was increased. Collectively, the results of IHC, WB and RT-PCR showed that PDGF-BB, PDGFR-β, and FGF-2 expressions peaked earlier in ginsenoside Rb1-treated rats than in model rats, consistent with the macroscopic observations.

**Conclusion:**

Collectively, these findings  indicated that ginsenoside Rb1 promotes burn wound healing via a mechanism possibly associated with upregulation of FGF-2/PDGF-BB/PDGFR-β gene and protein expressions.

## Background

Burns are common traumatic injuries whose depth is usually clinically classified according to a four-degree system [1]. Second-degree burns affect the epidermis and some parts of the dermis [2]. Therefore, second-degree burn wound healing is a complex multifactorial process involving inflammation, proliferation, re-epithelization, and remodeling phases [3, 4]. The stages overlap, and each involves the joint participation of diverse repair cells, inflammatory cells, growth factors, and extracellular matrix components, including fibroblast growth factor (FGF)-2, platelet-derived growth factor (PDGF), and other burn-related growth factors [5–7]. In the early stage of inflammation, macrophages promote the development of granulation tissue and secrete proinflammatory cytokines and growth factors such as interleukin-6 (IL-6), fibroblast growth factor (FGF), and PDGF, which regulate angiogenesis by affecting the proliferation, migration and differentiation of vascular endothelial cells [8]. During the proliferation and maturation stage, fibroblasts and myofibroblasts generate collagen and extracellular matrix components and form a bridge between the wound edges. Fibroblasts and their associated growth factors play a key role through out the process of wound repair [9]. Therefore, the expression levels of FGF-2 and PDGF-BB/PDGFR-β can accurately reflect the progression of wound healing, and these molecules are important targets for research on the effects of drugs on wound healing.

**Fig. 1:**
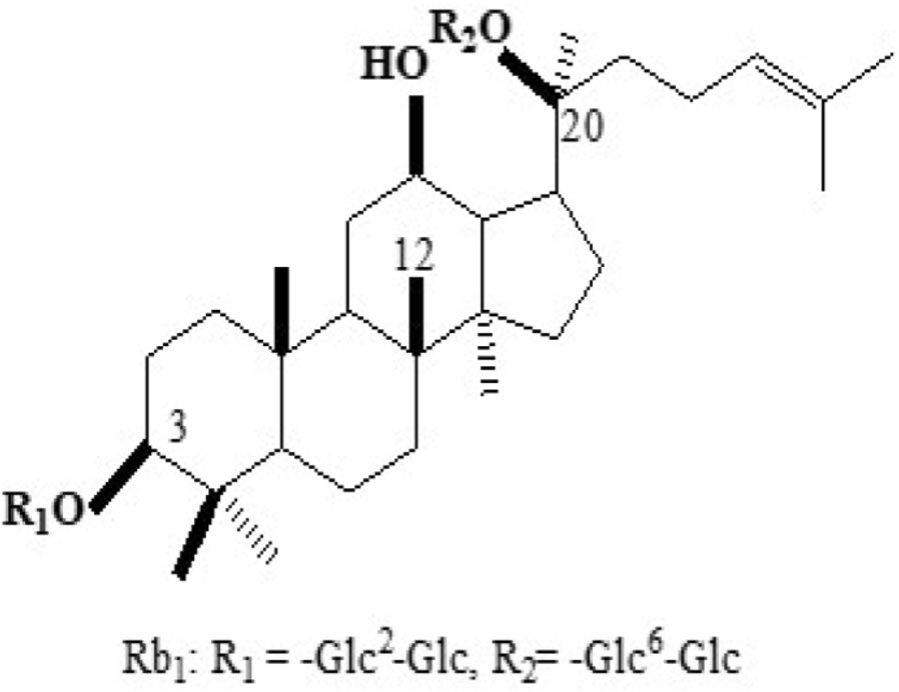
Chemical structure of ginsenoside Rb1

Currently, silver sulfadiazine (SSD) is commonly used to prevent burn wound infection and to relieve symptoms during the clinical treatment of second-degree burns [10]. However, given the adverse effects of these drugs (cytotoxicity, side effects related to antibacterial activity, etc.) [11], the prognosis of some individuals is still not optimistic. Consequently, local topical medications for the treatment of burns and scalds with clear medicinal effects and fewer adverse reactions than currently available agents are urgently needed. *Panax notoginseng*is a traditional herbal medicine that has been used therapeutically for cardiovascular diseases, inflammatory diseases and traumatic injuries as well as for external and internal bleeding due to injury [12]. Ginsenoside Rb1, the crucial active constituent of *P. notoginseng*, has attracted widespread attention because of its potential anti-inflammatory [13, 14], antioxidant and cell growth-promoting effects [15, 16]. In this study, the promotive effects of ginsenoside Rb1 on burn wound healing and mechanisms underlying these effects were explored to further contribute to a research foundation for the clinical application of ginsenoside Rb1 to treat burns and scalds (Fig. 1).

## Materials and methods

### Reagents and antibodies

Ginsenoside Rb1 (purity > 99.5%) was purchased from the National Institute for the Control of Pharmaceutical and Biological Products (Beijing, China). SSD cream (1%) was used as a positive control.Anti-FGF-2, anti-PDGFR-β, anti-PDGF-BB and anti-β-actin antibodies were purchased from Abcam (Shanghai, China). A BCA protein quantification kit was purchased from BiyunTian (Shanghai, China). Electrochemiluminescence (ECL) reagent was purchased from Millipore (Billerica, MA, USA). A total RNA extraction kit was purchased from Tiangen (Beijing, China). A RevertAid First Strand cDNA Synthesis Kit was purchased from Thermo Fisher Scientific (Waltham, MA, USA). RT-PCR Master Mix was purchased from Vazyme (Nanjing, China).The various other chemicals used were of analytical grade and commercial origin.

### Preparation of ginsenoside Rb1 ointment

To prepare 5 g/kg ginsenoside Rb1, 50 g of ginsenoside Rb1 was combined with 75 mL of 75% ethanol, and 2.5 g of sodium carboxymethyl cellulose and 5 g of Vaseline were then added. The mixture was brought to the necessary volume with distilled water and stirred continuously until a uniform high-dose ointment weighing 100 g formed. This ointment was refrigerated and set aside. The ointment had good spread ability, a uniform particle size distribution, and moderate viscosity. The doses of ginsenoside Rb1 used in the medium-dose and low-dose groups were 50% and 25% of that used in the high-dose group, respectively. The ointment applied to rats in pathological model (MD) group was prepared without ginsenoside Rb1.

### Animals

Male adult Sprague–Dawley rats (144 rats, 250–320 g) were randomly divided into a blank control group, a MD group, three treatment groups of ginsenoside Rb1 (1.25 [low-dose], 2.5 [medium-dose], and 5 [high-dose] g/kg), and a 1% SSD group. The rats were maintained on a 12 h/12 h light/dark cycle and had free access to food and water. All processes involving rats were authorized by the Animal Research Ethics Committee of Kunming Medical University (SYXK/2016–0004). The experiment was performed according to the 3Rs of animal use.

### Second-degree burn modeling

Rats were housed under standard laboratory conditions at 25 °C and supplied with food and water daily. The fur was shaved from the dorsal surface of each rat with electric clippers, and the skin was depilated with 20% Na_2_S (dissolved in alcohol) before induction of scald injury [17, 18]. After a 12 h fast, the rats were anesthetized with 3% pentobarbital (10 mg/kg body weight) by intraperitoneal (*i.p.*) injection. The rats were positioned in a premade template with a rectangular opening to expose the dorsal skin surface area and protect the remaining skin from direct exposure. The burn area was limited to approximately 2 cm × 4 cm with the template. A volume of 0.8 mL of mixed fuel (stock: gasoline, 25 mL; 95% alcohol, 60 mL; rosin, 60 g; glycerol, 5 mL; and xylene, 5 mL) was applied to each wound. The fuel was smeared evenly on the dorsal skin of each rat, lit with an open flame, and allowed to burn for 20 s. The fire was then extinguished as quickly as possible with a wet cloth. Pathological changes in the skin tissue were observed to confirm the injury depth. Based on histopathological analysis of skin slices, second-degree burns were characterized by damage to the epidermis and part of the superficial dermis, partial necrosis of tissue cells, and swelling and degeneration of some cells in the superficial dermis. After burn induction, the rats were housed separately. The wounds were bandaged and treated daily until completely healed. After 35 days of treatment, all rats were sacrificed.

### Sampling and treatment application

Samples of skin from the burn wounds were collected at the same time points on days 0, 7, 14, and 24 after burn injury. After sample collection, the wounds were sutured, smeared with erythromycin ointment and bandaged with sterile gauze. Furthermore, the rats were given drugs within 2 h after burn injury. In rats in the MD group, the second-degree burn wounds were not treated with medication but instead were treated with ointment lacking ginsenoside Rb1. Rats in the blank control group did not undergo modeling or treatment. In rats in the ginsenoside Rb1 groups, the wounds were evenly smeared with ginsenoside Rb1 ointment (1.25, 2.5, or 5 g/kg). In rats in the positive control group, the wounds were treated with 1% SSD (10 g/kg). The treatments were applied two times a day for 24 days at the same times each day. Before each application, any residual drug on the wounds was rinsed off with normal saline. After application, the wounds were bandaged with sterile gauze. The scab removal and fur growth times were recorded.

### Measurement of wound areas

The wound healing rates of the rats are expressed as wound closure rates. The area of each wound was traced, measured, and converted into a percentage, relative to the area of the wound at the time of injury, which was set as 100%. The wound areas were monitored every 7 days using a transparent graph sheet and a marker and were found to gradually decrease. The wound areas were measured with the design and drawing tools in Image-Pro Plus(Media Cybernetics Inc., USA, IPP6.0). The wound closure rate represents the percent reduction in the area of the original wound and was calculated as follows: wound closure rate (%) = (A0 − An)/A0 × 100, where A0 is the initial wound area and An is the wound area on day n.

### Hematoxylin–eosin (HE) staining

After the rat model of second-degree burn injury was successfully established, the skin samples were cut into four segments. One segment was used for HE staining: in brief, skin tissues were fixed with 4% formalin, decalcified, embedded, sectioned (at a thickness of 5 mm), and stained with HE. The second segment was fixed with 4% paraformaldehyde for immunohistochemistry (IHC), and the segmentpart was frozen for Western blotting (WB). The fourth segment was immediately immersed in RNA protective solution for analysis by real-time polymerase chain reaction (RT-PCR).

### IHC

For IHC, skin tissue was embedded in paraffin and sliced into 4-μm-thick serial histologicalsections. In brief, dewaxed tissue sections (four serial sections/skin sample) were processed for antigen retrieval in a pressure cooker in 0.01 M of citric acid salt buffer (pH 6.0). The sections were washed with phosphate-buffered saline (PBS) and were then placed in 5% normal goat serum diluted in PBS for 2 h at room temperature (22 °C–25 °C) to block nonspecific protein binding. After the serum was removed by washing, the sections were incubated with primary antibodies (specific for FGF-2[1:200], PDGF-BB[1:200], and PDGFR-β[1:300]) diluted in PBS overnight at 4 °C and were then stained with DAB. The sections were counterstained with hematoxylin, dehydrated, and sealed with neutral gum. The positively stained cells exhibited a brown cytoplasm with yellow precipitates. Six sections per rat were selected. Starting with the 5th serial section, every 10th serial section thereafter (i.e., the 15th, 25th, 35th, 45th, and 55th sections) were placed in a row (n = 6). Six nonoverlapping fields of view per section were randomly imaged under a light microscope at 400 × magnificationand analyzed using IPP6.0 software. The distributions of FGF-2, PDGF-BB and PDGFR-β in the skin tissues were observed.

### WB

Skin samples from rats in each group were homogenized with proteinase-containing protein extraction reagent, and the supernatant was collected. The protein concentrations in the tissues were tested with a protein assay kit. Supernatant samples containing 50 µg of tissue proteins were loaded and heated to 95 °C for 10 min, and the proteins were then separated by sodium dodecyl sulfate–polyacrylamide gel electrophoresis on 8% or 10% gels using a Mini-PROTEANII device. The protein were then electroblotted onto polyvinylidene difluoride membranes. The membranes were blocked in 5% BSA solution for 2 h and were then incubated with primary antibodies specific for FGF-2(1:2000), PDGF-BB(1:1500), PDGFR-β(1:2000), and β-actin(1:1500) overnight at 4 °C. After three rinses with TBST, the membranes were placed in secondary antibody solution (horseradish peroxidase-conjugated rabbit IgG [1:2000]) for 1 h and were then cleaned and developed with ECL reagent. A ChemiDoc XRS system was used to image the chemiluminescence signals, and ImageJ 1.4.3.67 software was used to quantify the protein band densities. The densities of individual protein bands were normalized to that of the corresponding β-actin band, and the results are presented in arbitrary units.

### RT-PCR analysis

Total RNA was extracted from skin samples using a TRIzol kit and was then reverse-transcribed using a Quantscript RT kit. PCR was performed using an ABI7900HT RT-PCR system (ABI Inc., USA) in accordance with the established methods. The 2^−ΔΔCT^ values were calculated to indicate the relative expression levels of four mRNAs (FGF-2, PDGF, PDGFR-β and β-actin) [19, 20]. Four PCR primers were selected randomly and used to analyze changes in the skin tissue. The thermal cycling conditions used for PCR were as follows: 35 cycles of denaturation at 95 °C for 10 s, annealing at 60 °C for 15 s, and elongation at 70 °C for 10 s. PCR was performed in triplicate using a 20-µL mixture containing 4 µL of 5 × FastPfu buffer, 2 µL of 2.5 mM dNTPs, 0.8 µL of each primer (5 µM), 0.4 µL of FastPfupolymerase, and 10 ng of template DNA. The primer sequences used to amplify the specific genes were as follows:

PDGF-BB(110 bp): (forward) 5'-CATCGAGCCAAGACACCTCA-3'. (reverse) 5'-AGTGCCTTCTTGTCATGGGT-3'. PDGFR-β(154 bp): (forward)5'-TGTCATGGGTAC-3'. (reverse) 5'-TGTCATGGGTAC-3'. FGF-2(115 bp): (forward) 5'-TATGTGGCACTGAAACGAAC-3'. (reverse) 5-'CTCAATGACCTGGCGAAGAC-3'. β-actin (115 bp): (forward) 5'-AGGCCAACCGTGAAAAGATG-3'. (reverse) 5'-ATGCCAGTGGTACGACCAGA-3'.

### Statistical analysis

The data are presented as the mean ± standard deviation (SD) values. We used the LSD test in the one-way ANOVA module of SPSS software to perform pairwise comparisons between multiple groups. Statistical graphs were generated with GraphPad Prism 7.0 software. A P value < 0.05 was considered to indicate statistical significance.

## Results

### Pathological observations and macroscopic appearance after burn injury

**Fig. 2:**
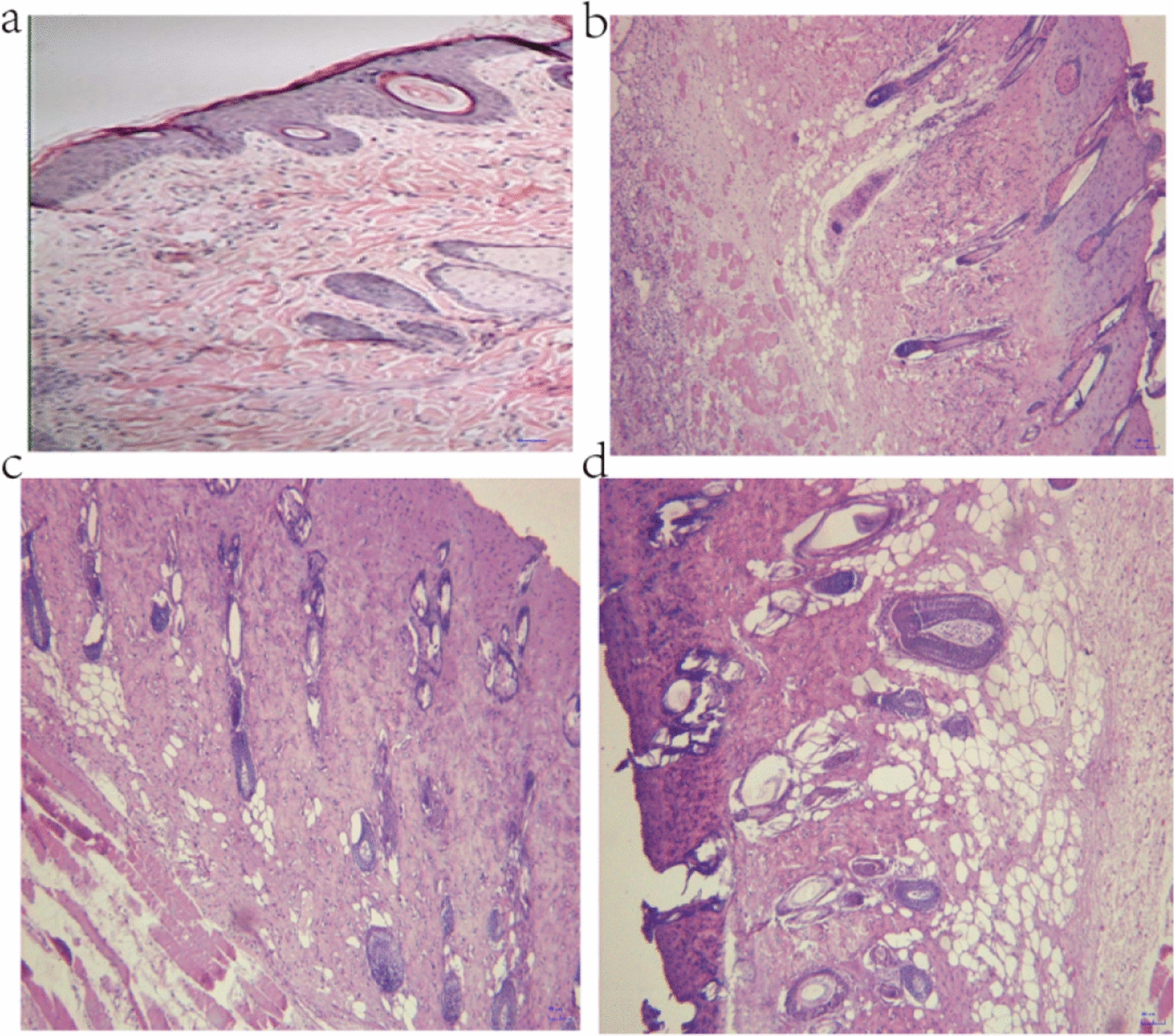
Pathological observations (HE staining, 100 ×). **a** Normal rat skin. **b**–**d** Second-degree burn wound tissue. Scale bars,100 μm. n = 6 rats per group

**Fig. 3:**
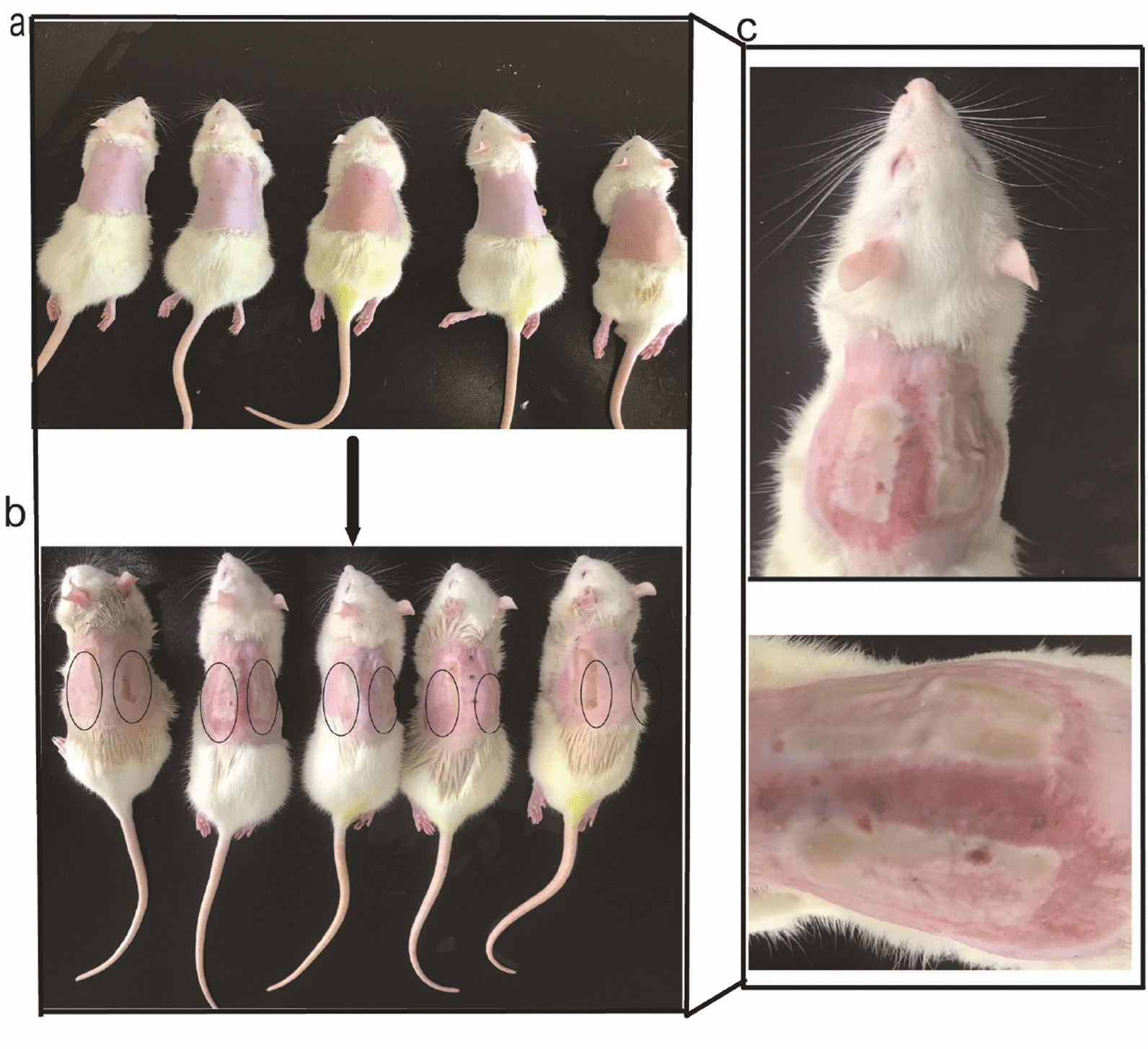
Macroscopic appearance of wounds in the second-degree burn model. **a** Anesthetized rats. **b** Location of the second-degree burn relative to the spine; the left and right sides were symmetrical. **c** Burn wounds. n = 6 rats per group

In the blank control group, the skin of the rats was normal and exhibited a clear structure, and the cells in the basal layer were cuboidal and neatly arranged. The epidermis and dermis were intact, no hyperemia or edema was found, and the cells of the skin appendages were viable. However, in the MD group, the structures of the epidermis and part of the dermis were partially damaged. Obvious edema, exudation of a large number of inflammatory cells, and necrosis of the superficial dermis were observed, although the cells of the skin appendages were viable. Thus, these rats showed typical manifestations of second-degree burns, as shown in Fig. 2. A single blister was observed on the skin surface of each second-degree burn model rat. In each rat, the base of the wound was flush, and the edge of the burn area was neat, exhibiting a typical clinical manifestation of second-degree burn injury, as shown in Fig. 3.

### Therapeutic effects of ginsenoside Rb1 on second-degree burn

**Fig. 4:**
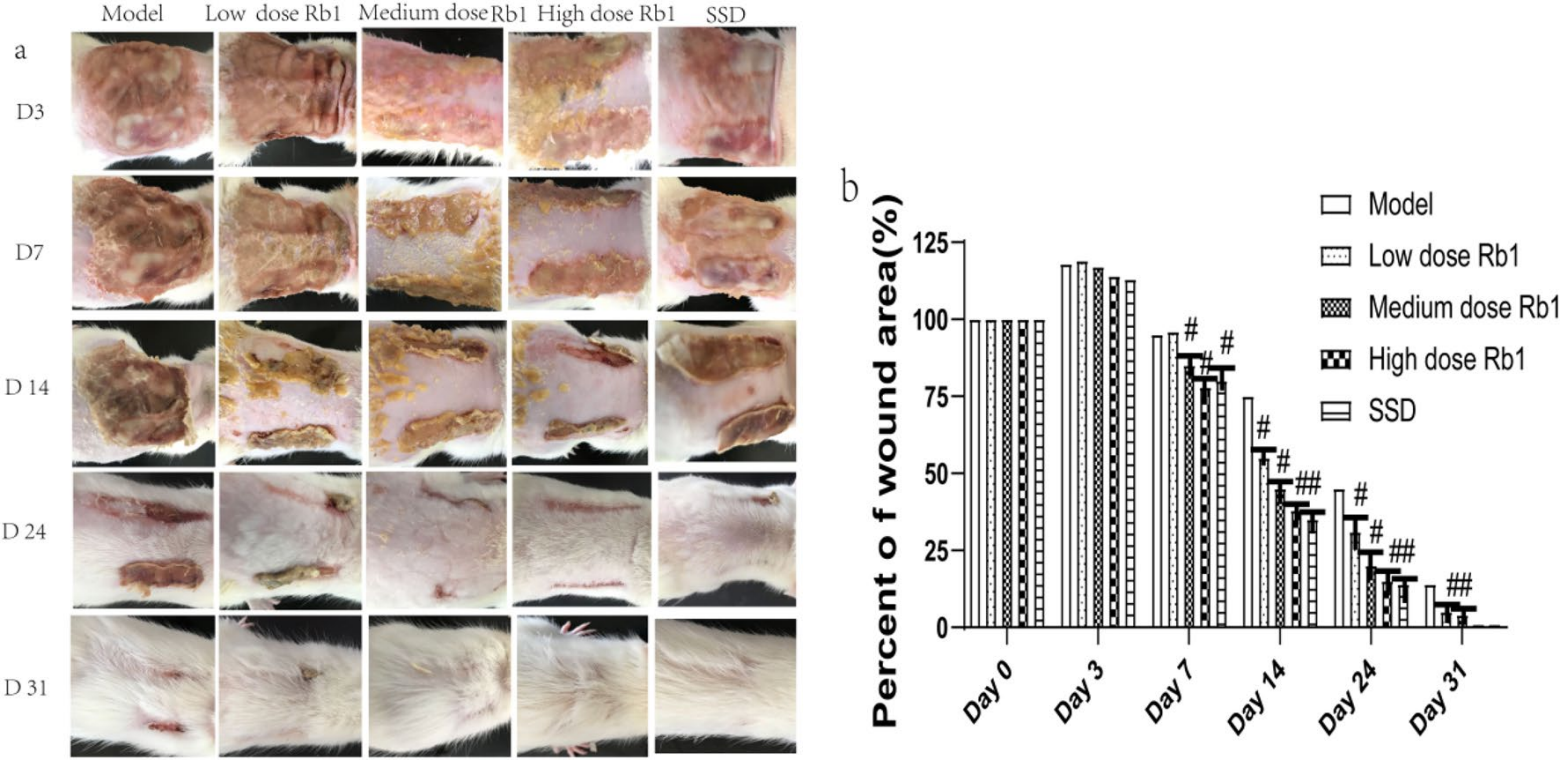
Macroscopic appearances and variations in the size of burn wounds. **a** Macroscopic appearance of the wound area on days 3, 7, 14, 24, and 31 in rats with or without high-dose ginsenoside Rb1 or 1% SSD treatment. **b** Bar graph of the quantified wound sizes on days 0, 3, 7, 14, 24 and 31. Each data value indicates the mean ± SD from six rats. The data are expressed as the mean ± SD values. ^#^P < 0.05 vs.the MD group on the same day. n = 6 rats per group

The macroscopic appearances and variations in the size of the burn wounds in ginsenoside Rb1-treated rats and rats subjected to other protocols are shown in Fig. 4. On day 0, the average wound area was 8.036 ± 0.125cm^2^ within 2 h after burn injury, and no significant differences in the primary wound area were identified among the 6 groups (P > 0.05). By day 3, the wound areas in all groups had increased. On day 7, the wound areas in all treatment groups was significantly smaller than that in the MD group (P < 0.05), possibly due to an accelerated wound healing process in the treatment groups, especially in the high-dose ginsenoside Rb1 group. These findings suggest that ginsenoside Rb1 has a significant positive effect on the wound closure rate. On day 14, the wound closure rate in the high-dose ginsenoside Rb1 group was significantly higher than that in the other groups (P < 0.01). By day 24, the rats in the high-dose ginsenoside Rb1 and SSD groups were not completely covered with fur, but the wound scabs were almost completely gone, and no significant differences in the skin and fur between the rats in these groups were observed. By day 31, both the skin and fur of the rats had recovered and were intact, as shown in Fig. 4.

### Changes in wound healing revealed by HE staining

**Fig. 5:**
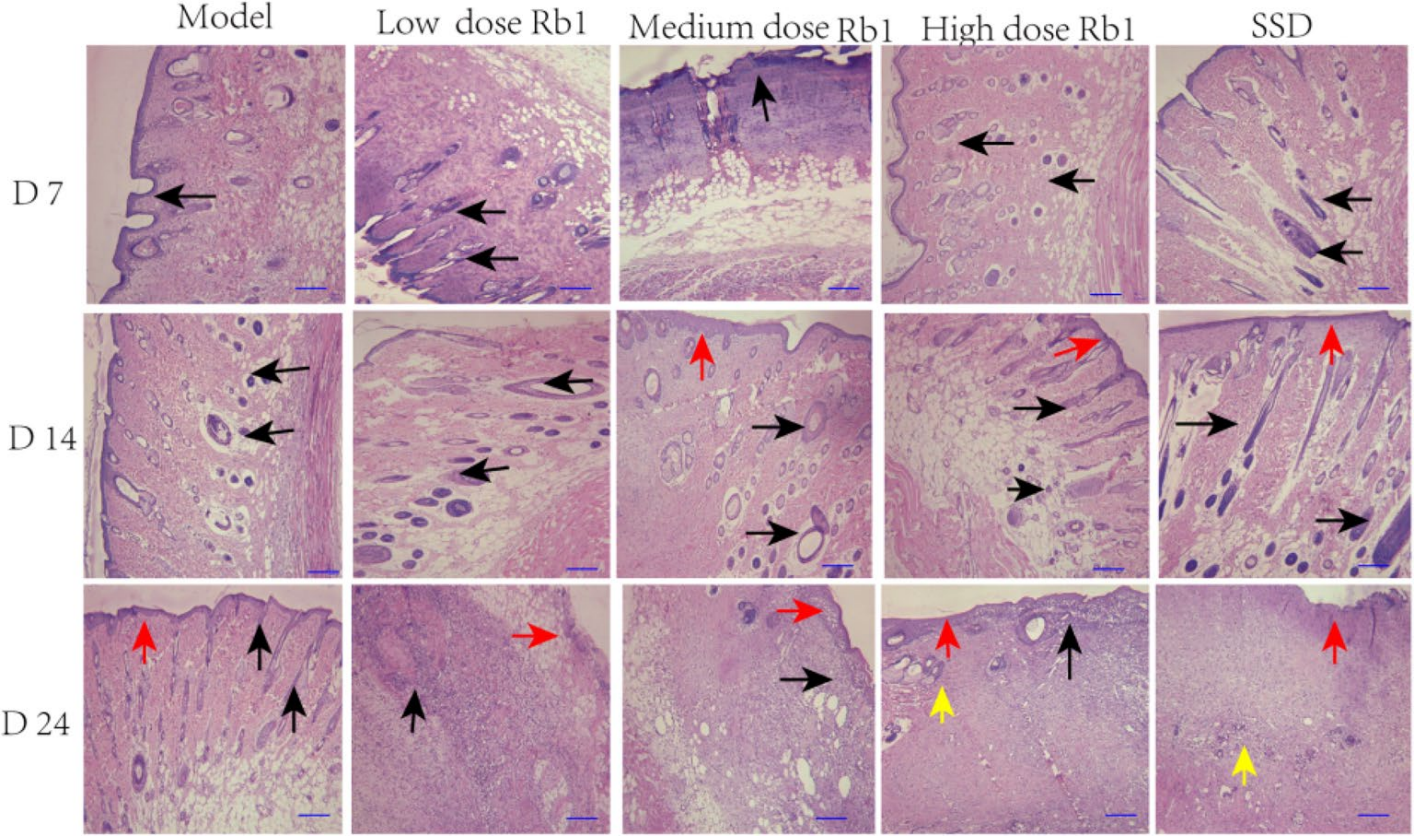
Effects of ginsenoside Rb1 on second-degree burn wound healing in rats (HE, 100 ×). Specimens were obtained from live rats on days 7, 14, and 24 after burn injury. The black arrows indicate recruitment of inflammatory cells to the wound area, the red arrows indicate re-epithelialization, and the yellow arrows indicate hair follicles. Scale bars, 100 μm. n = 6 rats per group

The therapeutic effects of ginsenoside Rb1 in the second-degree burn model were observed by microscopic examination of morphological features, as shown in Fig. 5. On day 7, the rats in the high-dose ginsenoside Rb1 and SSD groups exhibited greater inflammatory cell infiltration of the myometrium and greater hyperemia and edema of the epidermis and dermis than rats in the MD group. By day 14, in the MD group rats, part of the epidermis was necrotic, and a large number of inflammatory cells had infiltrated the muscular layer. The rats in the high-dose ginsenoside Rb1 and SSD groups exhibited decreased infiltration of inflammatory cells in the muscle layer, and obvious epithelialization of skin tissue. On day 24, the rats in the MD group still exhibited enhanced inflammatory cell infiltration on the surface of the necrotic epidermis and a small amount of granulation tissue proliferation; in addition, no obvious hair follicle repair was observed. However, the granulation tissue under the epidermis had proliferated and was obviously repaired; moreover, some epidermal tissue had been completely repaired. Well-repaired hair follicles were visible in rats in the high-dose ginsenoside Rb1 and SSD groups. Our results suggest that ginsenoside Rb1 treatment can accelerate reepithelialization, thus accelerating wound healing.

### Ginsenoside Rb1 promotes second-degree burn healing in model rats

The scab elimination and fur growth times were shorter in the ginsenoside Rb1 and SSD groups than in the MD group (P < 0.05). In addition, in the high-dose ginsenoside Rb1 and SSD groups, the wound healing rate was significantly increased, and the wound healing time was significantly reduced (P < 0.05). The results in the high-dose ginsenoside Rb1 and SSD groups were similar; no significant differences were found (P > 0.05) (Tables 1, 2).

**Table 1:** Comparison of differences in the scab removal and fur growth times among the groups ($\stackrel{-}{x}$±s)

Group	n	Scab removal time	Fur growth time
MD	24	25.60 ± 1.21	31.01 ± 2.80
SSD	24	21.32 ± 1.30^##^	24.10 ± 2.30^##^
Low-dose Rb1	24	23.10 ± 1.02	26.01 ± 3.51
Medium-dose Rb1	24	22.50 ± 1.25^#^	25.62 ± 1.52^#^
High-dose Rb1	24	21.01 ± 1.01^##^	24.01 ± 1.50^##^

^#^P < 0.05, and ^##^P < 0.01 compared with the MD group

**Table 2:** Comparison of cure rates among the groups at different time points ($\stackrel{-}{x}$±s) (%)

Group	Day 7	Day 14	Day 24	Day 31
MD	7.05 ± 24.12	56.00 ± 23.12	78.00 ± 3.01	93.02 ± 1.28
SSD	10.01 ± 27.40	86.10 ± 14.00^##^	89.1 0 ± 0.90^##^	99.98 ± 0.25^#^
Low-dose Rb1	7.80 ± 23.13	61.10 ± 13.14	75.12 ± 1.02	95.38 ± 0.92
medium-dose Rb1	8.80 ± 24.26	75.24 ± 15.23^#^	80.00 ± 0.81^#^	98.46 ± 0.82^#^
high-dose Rb1	9.26 ± 25.34	83.42 ± 12.22^##^	88.99 ± 0.52^##^	99.99 ± 0.71^#^

^#^P < 0.05 and ^##^P < 0.01 compared with the MD group

### Effects of ginsenoside Rb1 on FGF-2, PDGF-BB, and PDGFR-β protein expressions during wound healing

**Fig. 6:**
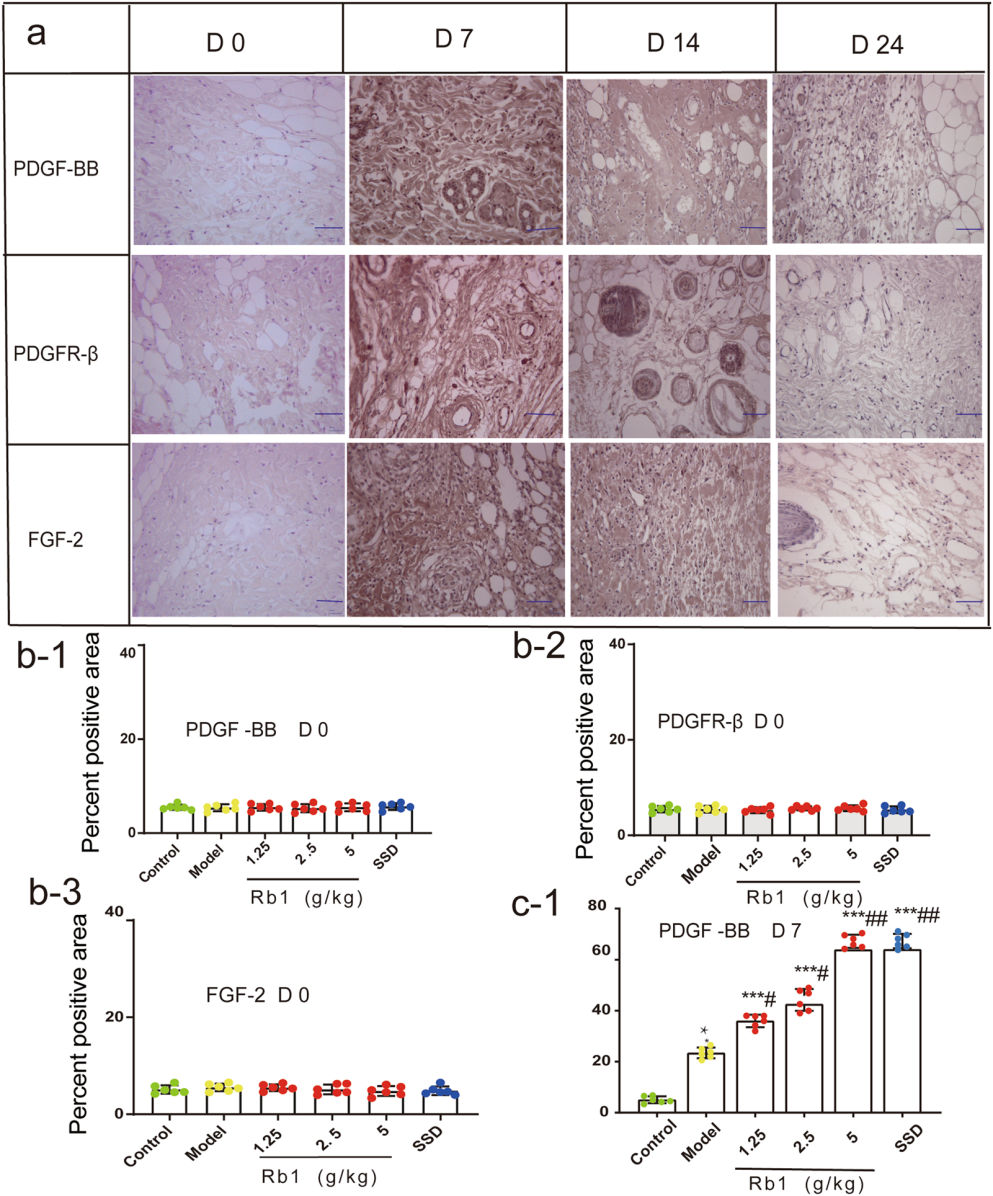

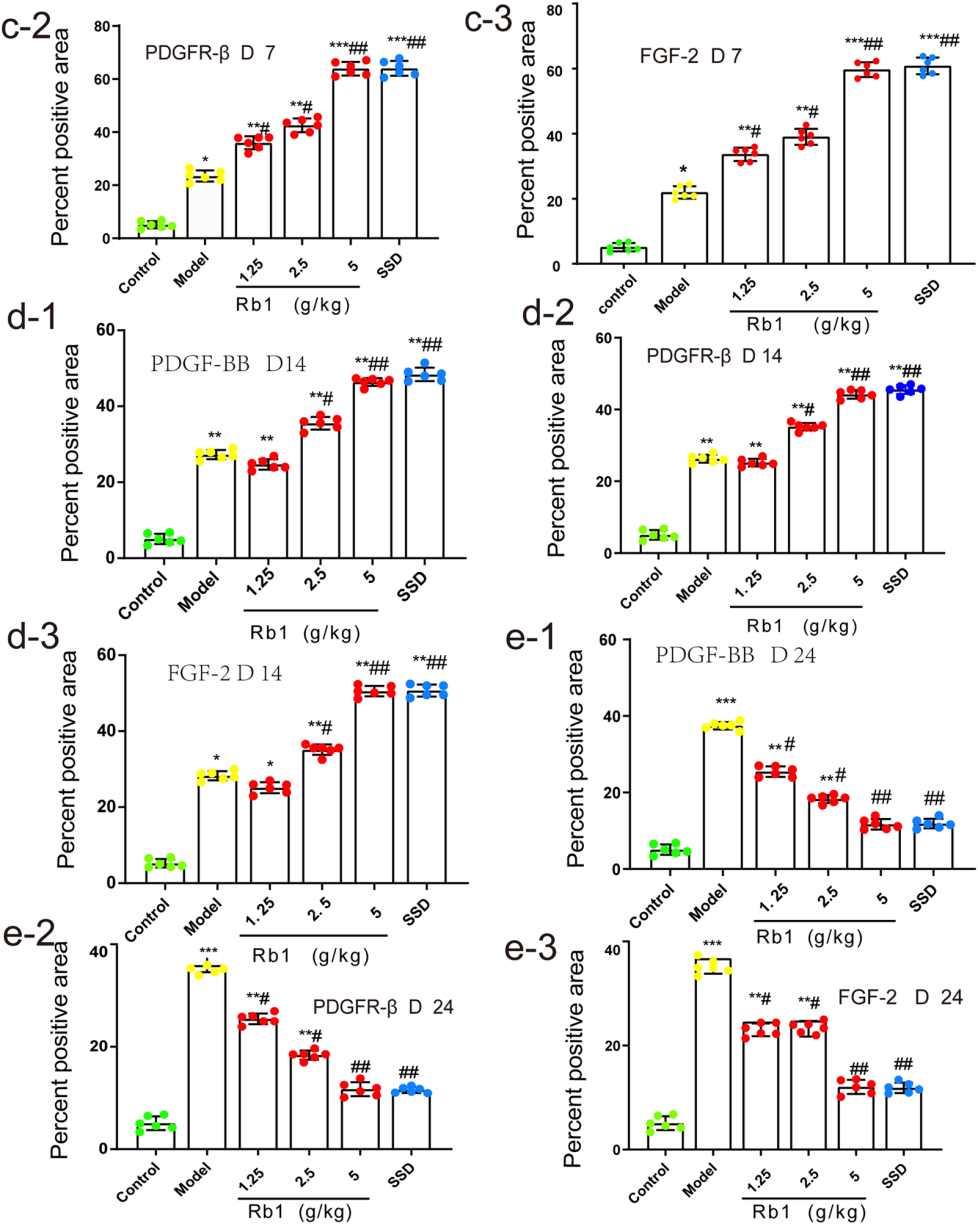
Detection of PDGF-BB, PDGFR-β and FGF-2 protein expressions during wound healing by IHC (400 ×). **a** Representative image of PDGF-BB, PDGFR-β, and FGF-2 protein expression in the high-dose ginsenoside Rb1 group at different time points. **b**–**e**, Statistical analysis of the percent positive area in each experimental group. The percent positive area was quantified with IPP6.0 software. The data are expressed as means ± SDs. Scale bars, 100 μm. *P < 0.05, **P < 0.01, and ***P < 0.001 vs. the control group; ^#^P < 0.05 and ^##^P < 0.01 vs.the MD group. n = 6 rats per group

**Fig. 7:**
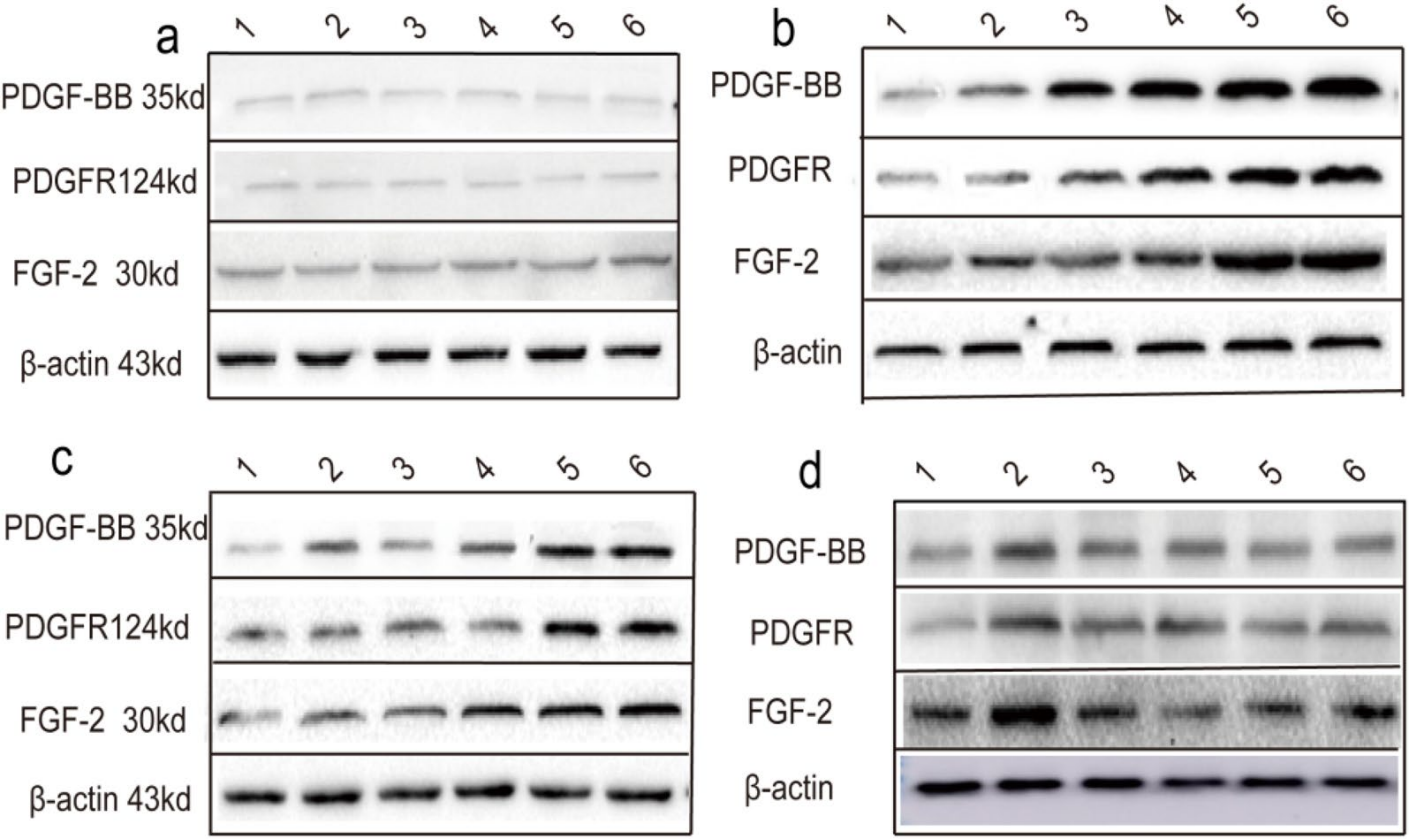

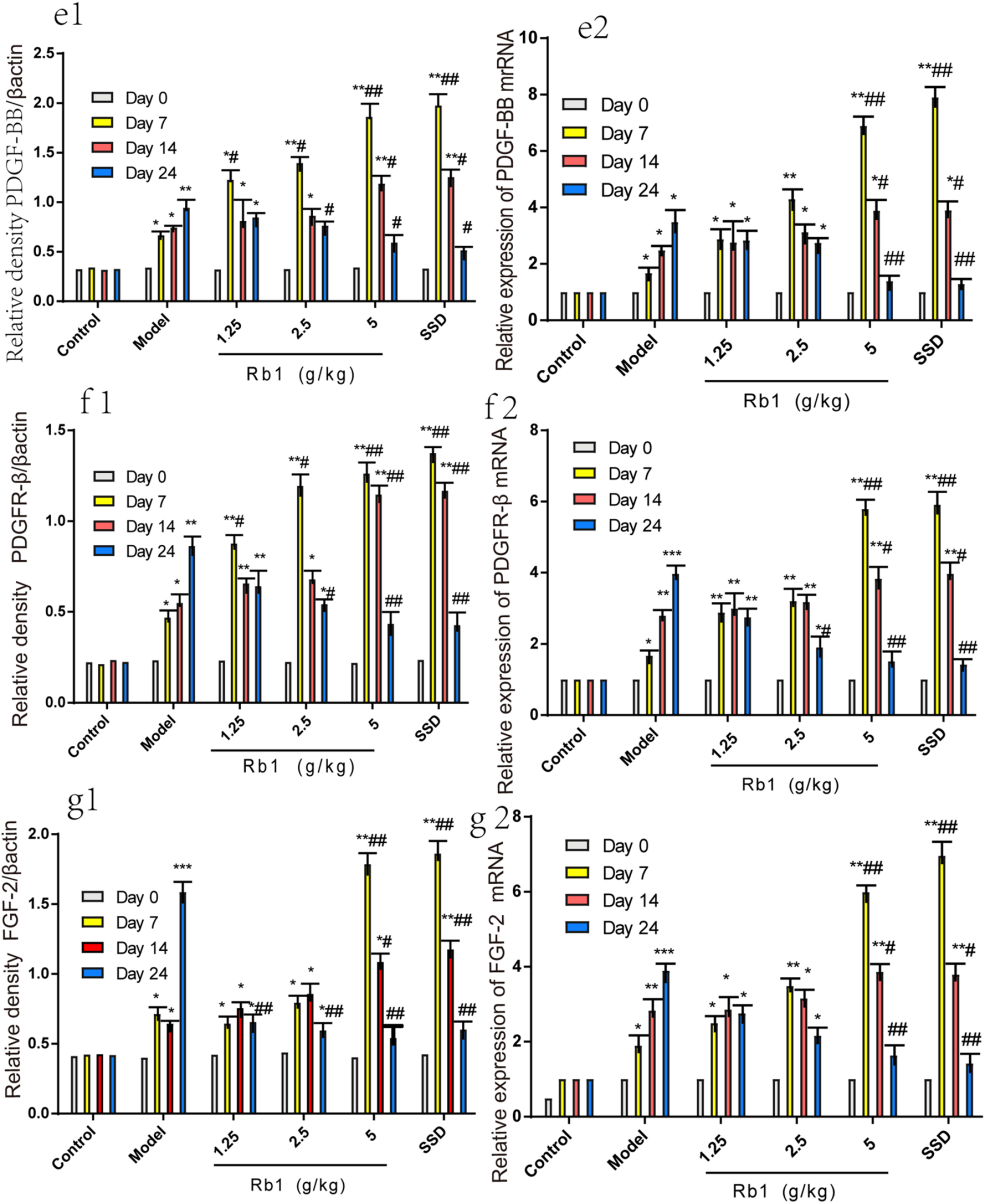
Effects of ginsenoside Rb1 on burn wound healing. **a**–**e** Expressions of PDGF-BB, PDGFR-β, and FGF-2 on days 0, 7, 14, and 24 after burn injury. The protein expression levels of PDGF-BB, PDGFR-β, and FGF-2 were normalized to those of β-actin and quantified with ImageJ v1.49 software. The data are expressed as the means ± SDs. *P < 0.05, ** P < 0.01, and *** P < 0.001 vs. the control group; ^#^P < 0.05 and ^##^P < 0.01 vs. the MD group. n = 6 rats per group. βactin was used as the internal reference. 1 Control group, 2 MD group, 3 Low-dose ginsenoside Rb1 group (1.25 g/kg), 4 Medium-dose ginsenoside Rb1 group (2.5 g/kg), 5 High-dose ginsenoside Rb1 group (5 g/kg), 6 Positive control group (1% SSD)

The results of IHC showed that the experimental groups exhibited positive PDGF-BB, PDGFR-β, and FGF-2 staining on day 0 and that there were no significant differences among these groups (P > 0.05). On day 7, unlike the blank control group, the experimental groups showed burn-induced positive brownish staining in epidermal and dermal skin tissues. Additionally, the protein expression levels of PDGF-BB, PDGFR-β, and FGF-2 were increased significantly to their peak values in the high-dose ginsenoside Rb1 and SSD groups and were higher than those in the MD group (P < 0.001). The expression levels of these proteins did not differ significantly between the high-dose ginsenoside Rb1 group and the SSD group (P > 0.05). In all three ginsenoside Rb1 groups, the protein expression levels of PDGF-BB, PDGFR-β, and FGF-2 increased in a dose-dependent manner, and the increase in the high-dose ginsenoside Rb1 group was significant. On day 14, the protein expression levels of PDGF-BB, PDGFR-β, and FGF-2 were increased significantly in the MD group compared with the blank control group (P < 0.01). In contrast, PDGF-BB, PDGFR-β, and FGF-2 protein expression in the high-dose ginsenoside Rb1 and SSD groups was maintained at a high level. However, the expression levels of these proteins showed a downward trend. On day 24, the PDGF-BB, PDGFR-β, and FGF-2 protein expression levels were significantly increased to their peak values in the MD group (P < 0.001). The protein expression levels of PDGF-BB, PDGFR-β, and FGF-2 in the high-dose ginsenoside Rb1 and SSD groups continued to decrease, but remained slightly higher than those in the control group (P > 0.05), as shown in Fig. 6. The results of IHC were consistent with those of WB and RT-PCR analysis, as shown in Fig. 7.

### Ginsenoside Rb1 accelerates burn wound healing

To explore the therapeutic effects of ginsenoside Rb1 on second-degree burn wound healing, we compared the changes in the protein and mRNA expression levels of PDGF-BB, PDGFR-β, and FGF-2 in wound tissues of different experimental groups at different time points. The expression levels of PDGF-BB, PDGFR-β, and FGF-2 in wound tissue were markedly increased to their peak values in the high-dose ginsenoside Rb1 group on day 7 after burn injury. However, the protein and mRNA expression of PDGF-BB, PDGFR-β, and FGF-2 increased slowly in the MD group, peaking on day 24. Our data suggest that ginsenoside Rb1 treatment accelerates the peaks in the protein and mRNA expression of PDGF-BB, PDGFR-β, and FGF-2, consistent with the results of HE staining and IHC. Moreover, our molecular results were essentially consistent with the decreases in the scab removal and fur growth times and the increase in the wound healing rate in this second-degree burn model, as shown in Fig. 7.

## Discussion

Burn wound healing is a complex process that results in structural and functional defects in numerous organ systems. Given its complexity and the involvement of multiple organs, burn injury cannot be replicated in vitro. In the past twenty years, numerous animal models of burn injury have been established to clarify the pathophysiological progression of burn injuries [23]. However, because of the limitations of the various burn models, the pathological progression of burn injuries in models and the pathological changes that occur in clinical burn patients differ considerably. Thus, suitable animal burn models that can be used to study burn injury and obtain clinically translatable findings are urgently needed. Through many experimental trials, we optimized the fuel burning time and established a model of second-degree burns inflicted with an open flame that faithfully recapitulates the pathological progression of burn injuries in human patients. In most patients, small-to medium-sized second-degree burns are clinically treated as acute skin injuries. The main topical antibacterial drugs used in modern burn medicine are SSD and its derivatives [24, 25]. Although they produce side effects, these drugs have long been the most widely used topical burn drugs in both domestic and foreign clinics, and their excellent therapeutic effects on burn wounds are universally acknowledged [26]. Therefore, in the present study, we selected SSD as the positive control drug to verify the reliability of the positive validation system. Indeed, our data showed that SSD was effective in healing second-degree burn wounds.

Furthermore, we found that the size of the burn wounds was increased in all experimental groups except the blank control group on day 3 after burn injury. Some previous studies have reported similar observations; the size increase may be related to low perfusion, microthrombosis, autophagy-induced cytokine activity and tissue degradation, which cause the edges of ulcers to expand [21, 22]. Beginning on day 7, the wound area in the treatment groups was significantly smaller than that in the MD group. On day 14, the wound closure rate in the high-dose ginsenoside Rb1 group was significantly higher than that in the other groups. This finding was consistent with the results of HE staining, which indicated that ginsenoside Rb1 can accelerate wound epithelialization. The observations on day 24 revealed that the fur growth time of the rats in the high-dose ginsenoside Rb1 group was obviously decreased and that the scabs were almost completely detached in these rats. Thus, it can be inferred that Rb1 has obvious therapeutic effects in this rat model of second-degree burn injury.

To further clarify the effectiveness of our treatment in healing burn wounds, the gene and protein expression levels of wound healing markers such as PDGF-BB, PDGFR-β, and FGF-2 were evaluated at different time points. A previous study showed that FGF-2 showed strong positive staining in scar tissue and that the staining was located mainly in fibroblasts and vascular endothelial cells [23]. In addition, PDGF in the arterial wall has been proposed to interact with FGF-2 to promote the proliferation of SMCs in a rat model of carotid filament injury [27], and FGF-2/PDGF has been shown to enhance the stability of atherosclerotic plaques [28]. Both FGF-2 and PDGF-BB can promote the proliferation of hMSCs and rMSCs [29]. Furthermore, FGF signaling is a downstream regulator of PDGF-BB and participates in the antiaging activity of ESC conditioned medium (ESC-CM) [30]. Many studies have shown that the expression of FGF-2 is increased in injured blood vessels and that PDGF upregulates the expression of FGF-2 in cultured vascular cells [31, 32]. Furthermore, PDGF-BB has been confirmed to induce SMCs proliferation and late activation of ERK [33]. PDGF-BB has been shown to upregulate the expression of the high-molecular-weight protein FGF-2 through a mechanism dependent on the kinase activity of ERK-1/2 [34, 35]. FGF-2 mediates the stability and activation of the modulated PDGF-BB/PDGFR-β signaling pathway to synchronize its activity with that of the PDGFBB/PDGFR mRNA signaling pathway[33, 36]. Scholars have also proposed that FGF-2 and PDGF-BB synergistically regulate the coverage of perivascular cells in tumor vessels [37]. Thus, there is a known interaction between PDGF-BB and FGF-2, and accumulating evidence shows that PDGF-BB and FGF-2 are tightly linked by mutual regulation of each other’s activity [27, 30, 35, 38].

Our study suggests that ginsenoside Rb1 intervention accelerates the peaking of PDGF-BB, PDGFR-β, and FGF-2 protein and mRNA expression, an event that is related to the promotion of wound healing and a decreased wound healing time. Many studies have shown that acute trauma can induce the expression of genes encoding active growth factors and their receptors in epithelial tissue cells at the site of injury. However, the expression of these genes is inhibited during wound healing [6, 8, 39]. Therefore, the expression and inhibition of growth factor genes reflect the existence of molecular biological mechanisms that regulate tissue regeneration and reconstruction. Based on the effects of ginsenoside Rb1 on the expression of PDGF-BB, PDGFR-β, and FGF-2 in wound tissue at different time points, it can be inferred that ginsenoside Rb1 may exert its therapeutic effects on wound healing by upregulating the expression of FGF-2 via modulation of the PDGF-BB/PDGFR-β signaling pathway. This upregulation may accelerate epithelialization during wound healing prior to inflammatory and cellular reactions and ultimately accelerate cutaneous wound healing. These results further confirm the basis underlying the success of traditional application of *P.notoginseng* rhizomesto treat trauma, infection and skin inflammation [40].

In this study, we monitored dynamic changes in PDGF-BB, PDGFR-β, and FGF-2 protein and mRNA expression during wound healing in second-degree burn model. Our findings suggested that the PDGF-BB, PDGFR-β, and FGF-2 expression levels peaked earlier in ginsenoside Rb1-treated rats than in model rats. The time points of accelerated epithelialization and the early inflammatory reaction were detected by HE staining, and the results were consistent with those of IHC, WB and RT-PCR. Macroscopic observation further demonstrated that the scab removal time and fur growth time were decreased and that the wound healing rate was increased in the ginsenoside Rb1 group. Therefore, our molecular bioassay data were consistent with our macroscopic observation data. Ginsenoside Rb1 showed strong anti-inflammatory effects and accelerated wound healing at each time point. Overall, ginsenoside Rb1 significantly promoted skin wound healing in this second-degree burn rat model, and the underlying mechanisms were closely associated with upregulation of FGF-2 via modulation of the PDGF-BB/PDGFR-β signaling pathway in wound tissue. These results help lay a research foundation for the clinical application of ginsenoside Rb1 in the treatment of burns or scalds.

## Conclusion

These results indicated that ginsenoside Rb1 promotes burn wound healing via a mechanism possibly associated with up regulation of FGF-2/PDGF-BB/PDGFR-β protein and gene expressions levels.

## Data Availability

The data used to support the findings of this study are available from the corresponding author upon request.

## Abbreviations

Control: Blank control
MD: Pathological model
SSD: Silver sulfadiazine
PBS: Phosphate-buffered saline
PDGF-BB: Platelet-derived growth factor-BB
PDGFR-β: Platelet-derived growth factor receptor-β
FGF-2: Fibroblast growth factor-2
IL-6: Interleukin-6
